# Determinants of short birth interval among child-bearing age women in the Gedeb Hasasa district of the West Arsi zone, Ethiopia

**DOI:** 10.3389/fmed.2023.1025111

**Published:** 2023-01-24

**Authors:** Tewodros Yosef, Degfachew Debela, Nigusie Shifera

**Affiliations:** ^1^School of Public Health, College of Medicine and Health Sciences, Mizan-Tepi University, Mizan Teferi, Ethiopia; ^2^Public Health Department, Ethiopian Public Health Institute, Harare Regional Health Bureau, Harar, Ethiopia

**Keywords:** short birth interval, child bearing age women, Gedeb Hasasa district, West Arsi zone, Ethiopia

## Abstract

**Background:**

Short birth intervals have been linked to higher rates of fetal loss, prenatal mortality, and poorer child survival. Therefore, for countries like Ethiopia that have a population policy intended at reducing fertility, understanding the level and factors influencing birth spacing is crucial in order to apply appropriate intervention. This study aimed to assess the prevalence and determinants of the short birth interval among child-bearing age women in the Gedeb Hasasa district of the West Arsi zone, Ethiopia.

**Methods:**

A community-based cross-sectional study was conducted from 20 July to 20 August 2018. A multistage sampling method was used. Face-to-face interviews were conducted to gather data. The collected data were entered into Epi Data version 3.1 and later exported to SPSS version 21 for analysis. Logistic regression was used to identify factors associated with the short birth interval. The level of significance was declared at a *p*-value of <0.05.

**Results:**

A total of 714 women participated, with a 98% response rate. The median birth interval length was 32 months. The prevalence of the short birth interval was 50.4%. After adjusting for confounding variables, being a rural resident [AOR = 2.50, 95% CI (1.52, 4.09)], having an illiterate husband [AOR = 4.14, 95% CI (2.15, 8.45)], breastfeeding duration for 7–12 months [AOR = 3.16, 95% CI (1.95, 5.13)] and 13–23 months [AOR = 2.45, 95% CI (1.52, 3.95)], sex of the prior child [AOR = 0.63, 95% CI (0.45, 0.88)], and previous child alive [AOR = 0.20, 95% CI (0.14, 0.96)] were the determinants of short birth interval.

**Conclusion and recommendation:**

One in every two women practiced short birth intervals. The median birth interval duration was 32 months, which is below the minimum standard recommended by the WHO duration for the birth interval, which is 33 months. Short birth intervals were determined independently by residence, husband education, breastfeeding time, previous child’s sex, and previous child’s survival. Therefore, increasing women’s awareness of the ideal birth interval should be done through community health professionals and health developmental armies.

## Introduction

The time between two consecutive births is known as the birth interval (1, 2). The World Health Organization (WHO) advises a gestational gap of at least 33 months between two subsequent live births. A birth interval of less than 33 months is viewed as short, while the ideal birth interval is thought to be between 36 and 59 months (3). In addition, the U.S. Agency for International Development (USAID) stated that birth intervals of 3–5 years are preferable (4). A resting period between pregnancies that gives the mother time to recover from pregnancy, childbirth, and breastfeeding is referred to as optimal birth spacing (5). The ideal birth interval increases a child’s chance of surviving, and it gives women a chance to get healthier (3, 6, 7).

Short birth interval is a global public health problem that remains challenging in Ethiopia (8). Short birth intervals have been associated with adverse health outcomes, including infant, child, and maternal mortality (1, 9–11). The number of women who died during pregnancy- and childbirth-related causes was estimated to be 289,000 in 2013 or approximately 800 per day. Developing countries account for approximately 99% of all maternal fatalities (12).

A large multi-country study was conducted to view the association between inter-pregnancy interval and prenatal outcomes across Latin America from 1990 to 2009. It indicated that birth intervals of <12 and >2 months are associated with pre-eclampsia, neonatal mortality, and preterm birth, but not with other maternal or offspring outcomes (13). A systematic review and meta-analysis revealed that birth interval had a significant association with infant death. The pooled estimate odds ratio for an infant death with a birth interval of less than 24 months was determined to be 2.03 (14).

Women in low- and middle-income nations are more likely to have a short pregnancy and birth interval (15). An important indication of socioeconomic progress and a primary predictor of fertility in a country with a high population is the birth interval (16). With a total fertility rate of 4.6 (2.3 for urban and 5.2 for rural areas), Ethiopia has Africa’s second-highest population (17). The level of birth spacing and the factors influencing it must, therefore, be understood to implement the proper intervention in countries like Ethiopia where the population policy aims to reduce fertility (18). To the best of our knowledge, no research has been done in the target area. Therefore, using the WHO’s standard categorization for the birth spacing duration (3), this study sought to evaluate the level of short birth interval and its contributing factors among women of reproductive age who gave birth 5 years before this survey.

## Materials and methods

### Study design, area, and period

A community-based cross-sectional study was conducted in the Gedeb Hasasa district, West Arsi Zone of Southeast Ethiopia from 20 July to 20 August 2018. Hasasa is located at a distance of 284 km from Addis Ababa, the capital city of Ethiopia, and 87 km on the way from Shashamane, the capital of the west Arsi zone. The district has 20 kebeles. According to the 2007 EDHS report, the total population of the district and the number of women of child-bearing age were 254,367 and 56,291, respectively (17). The district is home to eight government health facilities, four non-profit clinics, 23 private clinics, and 11 drug stores. In the district, there was 50 health extension personnel.

### Populations

The source population was all women who had at least two consecutive live births in the previous 5 years. The study population was randomly selected, women who had at least two consecutive live births in the previous 5 years in the study area. Women with serious mental illness and hearing impairment were excluded.

### Sample size determination

The bigger sample size was employed for this investigation, and the sample sizes for each objective were computed as follows: For the first objective, a single population proportion formula was used to determine the sample size by making the following assumptions: The level of confidence was 95% (Z/2) = 1.96, marginal error (d) = 0.05, and a single population proportion (*p*-value = 0.57) were used as a percentage of child-bearing age women practicing short birth spacing from a cross-sectional study conducted in southern Ethiopia (7). After taking into account the 10% non-respondent rate and the fact that the sampling technique involves multiple stages of sampling, a design effect of 1.5 was taken into account, leading to a final sample size of 622. For the second specific objective, the sample size was calculated using the double population proportion formula using Epi-Info version 7 using an assumption of confidence level was 95%, power of 80%, ratio of 1:1, and by using a variable which has a significant association with dependent variable from the previous study, contraceptive use (the% of outcome in exposed was 51.5% and the% of outcome in unexposed was 65.1%) gives the largest sample size of all other variables (7), which was 440. The ultimate sample size was 726 after accounting for the design effect of 1.5 and the 10% non-respondent rate. Thus, 726 people made up the study’s ultimate sample size.

### Sampling procedure

A multistage sampling technique was used. Of the 20 kebeles, 30% (6 kebeles) of the districts were chosen by lottery to serve as the study’s representative sample. In each of the chosen kebeles, houses that met the requirements for inclusion were labeled. Households, where eligible women were discovered, were identified by a code. Systematic sampling procedures were then used to determine the skip interval, which was calculated as 12,542/726 = 17 by proportionally allocating sample size to each chosen kebeles. If there were two or more suitable women living in the same household, one of them was chosen by lottery. If no other eligible women could be identified in the household chosen, we moved on to the next family. For participants who were not present at the time of data collection, at least three visits were made to trace them.

### Study variables

The dependent variable was a short birth interval. The sociodemographic characteristics (age, age at marriage, occupation, religion, age at last pregnancy, ethnicity, and educational status of woman and her husband), obstetric history (parity, sex of index child, age at first birth, survival status of index child, family planning use, breastfeeding, EBF, and overall feeding duration), and place of delivery were the independent variables.

### Operational definitions

Birth interval is the time duration/period between two recent consecutive live births measured in months (1, 2). A short birth interval is a birth interval of less than 33 months after the preceding live birth (3). Optimal birth interval is referred to a birth interval of 33 months and above between the birth of the last child under study and the immediately preceding live birth (3). The index child is the first child of the recent two births (3).

### Data collection tools and procedure

Based on the examined literature and information gained from relevant studies, a structured questionnaire was prepared for data collection. The primary language in the study area, Afan Oromo, was used to collect the data. The questionnaire asks about the sociodemographics of the woman and her husband, their history of pregnancies, their family planning preferences, their nursing practices, and their awareness of the time between pregnancies. Cards for vaccinations were utilized to ascertain the dates of birth of the kids. We used maternal recall for those who had not received vaccinations, and community-based health extension workers were interviewed. After providing the appropriate information to the study participants and obtaining consent, face-to-face interviews with eligible women using a standardized questionnaire with closed-ended questions were undertaken. The first six health extension workers were chosen from their respective kebeles and instructed by the principal to label families and collect data. Health extension workers were taken into consideration since they are working closely with the community, particularly women, and because they have a deeper knowledge of household arrangements in the study area. Following the completion of the home labeling, six female nurses with a diploma who were proficient in both Afan Amara and Afan Oromo were sought out. For 2 days, the primary investigators trained them on the study’s goals, how to choose families, how to conduct interviews, how to obtain consent, and how to handle data.

### Data quality assurance

To confirm the quality of the data, a pretest was conducted on 5% of the sample size from the nearby district (Dodola district), which has a setting similar to the research area. Based on the results of the pretest, the necessary modifications were made. Training on the objectives of the study, the questionnaire’s questions, how to help study participants, and how to get respondents’ consent was provided to facilitators and data collectors for 2 days. The purpose of the study, its benefits, and the confidentiality of the information collected were all explicitly explained to study participants, allowing them to freely answer the questionnaire. The primary investigator, three BSc nurses, and a health extension worker in each kebeles monitored the fieldwork, and the principal investigators and the extension worker in each kebeles verified the collected data constantly on a daily basis for completeness.

### Data processing and analysis

Data were initially entered into Epi Data version 3.1 and exported to SPSS version 21 for additional analysis. Frequency distribution, mean, and median were computed as descriptive statistics. First, bivariate analyses were performed to evaluate the relationship between dependent and independent variables. For multivariate logistic regression analysis, variables were suitable if their associations with the dependent variables had a *p*-value of less than 0.25. The Hosmer–Lemeshow test of goodness-of-fit, which considers a good fit at a *p*-value of >0.05, was utilized to assess the goodness-of-fit of the final model. Finally, independent variables associated with the short birth interval were identified using a *p*-value of less than 0.05, and the strength of the association was evaluated using AORs with 95% confidence intervals.

## Results

### Sociodemographic characteristics

A total of 714 eligible women participated with a response rate of 98%. Of the 714 respondents participated, 600(84%) were rural residents. Nearly three-fourths of the respondents (74%) were women with no formal education, and 80% of them were homemakers in occupation. More than one-fourth (27.8%) of the respondents were married at the age of <18 (Table 1).

**TABLE 1:** Sociodemographic characteristics of the respondents in the Gedeb Hasasa district of the West Arsi zone, Ethiopia (*N* = 714).

Variables	Categories	Frequency (*n*)	Percentage (%)
Place of residence	Urban	114	16
	Rural	600	84
Religion	Muslims	524	73.4
	Orthodox	174	24.4
	Others*	16	2.2
Ethnicity	Oromo	658	92.2
	Amara	38	5.3
	Others**	18	2.5
Age of mothers	20–24	100	14
	25–29	285	39.9
	30–34	131	18.3
	35–39	124	17.4
	40–44	44	6.2
	Others***	30	4.2
Education of the mother	Illiterate	180	25.2
	Able to read and write	347	48.6
	Elementary (1–8)	143	20
	Secondary	22	3.1
	Tertiary	22	3.1
Education of the husband	Illiterate	150	21.0
	Able to read and write	38	5.3
	Elementary (1–8)	270	37.8
	Secondary (9–12)	158	22.1
	Tertiary (+12)	98	13.7
Occupation of the mother	Employee (GO/NGO)	46	6.4
	Housewife	570	79.8
	Merchant	60	8.4
	Others****	38	5.3
Occupation of the husband	Employee (GO/NGO)	74	10.4
	Housewife	52	7.3
	Merchant	26	3.6
	Farmer	476	66.7
	Student	80	11.2
	Daily worker	6	0.8
Maternal age during a marriage	<18	198	27.8
	≥18	516	72.2

*Protestant and Catholic; **Gurage and Silte; ***aged 15–19 and 45–49; ****merchant, farmer, and daily worker; GO, government organization; NGO, non-governmental organization.

### Knowledge of birth spacing

A total of 672 (94.1%) of the respondents had heard about the optimum birth interval between two successive live births. Of 672 respondents who had awareness about optimum birth interval time, 250 (35%) of them responded “to be below 3 years,” 350 (49%) of the respondents responded to be within 3–5 years, and the rest (16%) were believed to be greater than 60 months. The majorities, 679 (95.1%), believed that the short birth interval had a negative impact on both maternal and child health.

### Obstetric history and practice of birth spacing

At the time of the data collection period, 337 (47.2%) of the women had five children and more. A total of 664 (93%) women were required to have additional children. Of those who required additional children, 38.1% of them wanted to become pregnant then and the rest 61.9% desired to delay the pregnancy to sometime later. A total of 360 (50.4%) of the study participants practiced short birth intervals and the rest practiced above the minimum recommended standard by the WHO duration for the birth interval, which is 33 months. The median duration of the actual birth interval was 32 months. Whereas, the median duration of preferred birth interval for mothers was 36 months for the last two succeeding births.

### Breastfeeding, knowledge, and practice of modern contraception

Regarding the practice of breastfeeding, 98% of women breastfed their children and the rest of them fed formula milk. A total of 379 (53.1%) of the respondents breastfed their child for 13–23 months, and 506 (70.9%) stopped breastfeeding while the child was 24 months. The median duration of breastfeeding was 18 months. In total, 680 (95.2%) of the respondents were aware of the presence of modern contraceptive methods that help to delay or avoid pregnancy. Of 680 respondents, 676 (99.4%) of the respondents used contraceptives for birth spacing. The most preferred methods of contraception were injections accounting for 54.7%, followed by pills (24.9%).

### Determinants of short birth interval

After adjusting for confounding variables, being a rural resident [AOR = 2.50, 95% CI (1.52, 4.09)], having an illiterate husband [AOR = 4.14, 95% CI (2.15, 8.45)], breastfeeding duration for 7–12 months [AOR = 3.16, 95% CI (1.95, 5.13)] and 13–23 months [AOR = 2.45, 95% CI (1.52, 3.95)], sex of the prior child [AOR = 0.63, 95% CI (0.45, 0.88)], and previous child alive [AOR = 0.20, 95% CI (0.14, 0.96)] were the determinants of short birth interval (Table 2).

**TABLE 2:** Factors associated with short birth interval among child bearing age women in the Gedeb Hasasa district of the West Arsi zone, Ethiopia (*N* = 714).

Variables	Categories	Short birth interval		COR (95% C.I.)	AOR (95% C.I.)	*P*-value
		Yes	No			
Place of residence	Urban	46	68	1	1
	Rural	314	286	1.62 (1.08, 2.44)**	2.50 (1.52, 4.09)	**<0.001**
Age of mother	20–24	60	40	1.13 (0.44, 2.30)*	0.80 (0.29, 2.26)	0.679
	25–29	125	160	0.49 (0.24, 1.12)*	0.38 (0.15, 1.00)	0.050
	30–34	75	56	1.00 (0.40, 2.00)*	0.67 (0.25, 1.78)	0.418
	35–39	66	58	0.85 (0.34, 1.71)*	0.61 (0.23, 1.62)	0.319
	40–44	16	28	0.43 (0.15, 0.99)**	0.21 (0.07, 0.64)	0.060
	Others	18	12	1	1
Age at marriage	<18	116	82	1.58 (1.13, 2.20)**	1.20 (0.81, 1.78)	0.371
	≥18	244	272	1	1
Husband education	Illiterates	121	113	0.77 (0.47, 1.27)*	4.14 (2.15, 8.45)	**<0.001**
	Read and write	14	24	0.42 (0.19, 0.92)**	0.41 (0.17, 1.98)	0.055
	Primary (1–8)	129	103	0.90 (0.55, 1.49)*	0.93 (0.53, 1.64)	0.803
	Secondary (9–12)	46	78	0.43 (0.24, 0.75)**	0.39 (0.21, 2.72)	0.062
	Tertiary (12+)	50	36	1	1
Previous child sex	Male	198	223	0.72 (0.53, 0.97)**	0.63 (0.45, 0.88)	**0.007**
	Female	162	131	1	1
Previous child alive	Yes	348	352	0.17 (0.04, 0.74)**	0.20 (0.14, 0.96)	**0.044**
	No	12	2	1	1
Breastfeeding duration	0–6	4	10	0.56 (0.17, 1.82)*	1.47 (0.37, 5.77)	0.584
	7–12	98	42	3.28 (2.18, 4.94)**	3.16 (1.95, 5.13)	**<0.001**
	13–23	78	52	2.11 (1.41, 3.14)**	2.45 (1.52, 3.95)	**<0.001**
	≥24	178	250	1	1

AOR, adjusted odds ratio; CI, confidence interval; COR, crude odds ratio; *a *p*-value of <0.25, **a *p*-value of <0.05. The bold values show the level of significance. All bold values are variables significantly associated with the outcome variable (short birth interval).

## Discussion

To lower the frequency of unfavorable pregnancy outcomes, the World Health Organization suggested a minimum of 33 months between consecutive live births. Poor mother and child health outcomes, such as low birth weight, stillbirth, uterine rupture, neonatal death, maternal mortality, child malnutrition, and maternal hemorrhage, are linked to poorly spaced pregnancies (19). This study aimed to assess the prevalence and determinants of the short birth interval among child-bearing age women in the Gedeb Hasasa district, West Arsi zone, Ethiopia.

As a result, the median birth interval duration was 32 months, which is shorter than the minimal birth-to-birth interval which is advised to be 33 months (3). It was higher than 22 months in Uganda (20). It was lower than 38 months in Ethiopia (19). The prevalence of the short birth interval was 50.4%, 95% CI (46.7–54.1%). This finding was consistent with 52.4% in Uganda (20), 49.7% in Ghana (21), and 51.2% in Ethiopia (5). This finding was higher than 30.2% in Chad and 27.1% in the Democratic Republic of the Congo (22), 43.4 and 46% in studies in Ethiopia (23, 24), and 28.5% in Iran (1). It was lower than 56 and 57% of studies in Ethiopia (7, 25). The variation observed between this and other studies could be due to the difference in the sample size used, and the operational definition used (this study used <33 months cutoff point to say short birth interval, while some previous studies used <24 cutoff point). In addition, the sociodemographic, behavioral, and cultural factors differ resulting in significant variation.

Birth interval length was linked to the husband’s educational status. In this study, women with illiterate husbands were four times more likely than those with educated husbands to practice short birth intervals. This finding was consistent with studies (24–28). This is because educated husbands were more likely to cooperate with their wives in using family planning methods and value the importance of birth spacing.

Place of residence is found to be linked with birth interval duration. Women who reside in rural areas are 2.5 times more likely to have short birth intervals than those who reside in urban areas. This finding was supported by studies in Ethiopia (5, 7, 19, 21, 26). This could be associated with poor awareness of optimal birth intervals. This outcome may be attributable to enhanced social services and increased access to knowledge, possibilities for education, and work in urban than rural areas. However, other studies revealed that being an urban dweller is associated with a short birth interval (24, 28).

The duration of breastfeeding was significantly associated with birth interval. Women who breastfed for 7–12 months and 13–23 months had 3.2 and 2.5 times more likely to have a short birth interval compared with those who breastfed for more than 24 months. This finding was supported by the following studies (5–7, 15, 23, 25, 29–31), which revealed that the chance of a short birth interval was higher among women who breastfed for less than 24 months. This could be due to the contraceptive property of breastfeeding (32). The more you breastfed a child the more protected from becoming pregnant for a long time.

The sex of the previous child was statistically associated with the duration of the birth interval. Women who had a previous male child were 37% less likely to have a short birth interval than those who had a female child. This finding was supported by the following studies (6, 15, 23, 25, 30, 31, 33), which revealed that women who had a previous female child were more likely to practice the short birth interval. This may be due to the preference of having a male child if their previous child was female.

The life of the previous child was significantly associated with the duration of the birth interval. A woman whose previous child was alive was 80% less likely to have a short birth interval than a woman whose previous child died. This finding was supported by the following studies (25, 27, 28, 33–35), which revealed that mothers whose index child died were more likely to have a short birth interval than their counterparts. This could be due to the parent’s wish to replace a deceased kid as quickly as possible. In addition, the woman’s protection from lactation amenorrhea is less likely if the infant passed away.

### Limitations of the study

Recall bias may underestimate or overestimate the study findings (e.g., asking women to remember the duration of breastfeeding). The cross-sectional nature of the study makes it difficult to demonstrate cause-and-effect linkages between the dependent and independent variables.

## Conclusion and recommendation

One in every two women practiced short birth intervals. The median birth interval duration was 32 months, which is below the minimum standard recommended by the WHO duration for the birth interval, which is 33 months. Short birth intervals were determined independently by residence, husband education, breastfeeding time, previous child’s sex, and previous child’s survival. Therefore, increasing women’s awareness of the ideal birth interval should be done through community health professionals and the health developmental army.

## Data availability statement

The raw data supporting the conclusions of this article will be made available by the authors, without undue reservation.

## Ethics statement

The studies involving human participants were reviewed and approved by the Institutional Review Board (IRB) of Arsi University. The patients/participants provided their written informed consent to participate in this study.

## Author contributions

TY, DD, and NS contributed to the idea and design of the study, collection, analysis, and interpretation of data, writing and revision of the manuscript, the decision to submit it to the current journal, the final approval of the manuscript that will be published, and they also agreed to be responsible for all aspects of the work. All authors contributed to the article and approved the submitted version.
